# Aging of the Hematopoietic Stem Cell Niche: New Tools to Answer an Old Question

**DOI:** 10.3389/fimmu.2021.738204

**Published:** 2021-11-11

**Authors:** Francesca Matteini, Medhanie A. Mulaw, M. Carolina Florian

**Affiliations:** ^1^ Stem Cell Aging Group, Regenerative Medicine Program, The Bellvitge Institute for Biomedical Research (IDIBELL), Barcelona, Spain; ^2^ Program for Advancing the Clinical Translation of Regenerative Medicine of Catalonia, P-CMR[C], Barcelona, Spain; ^3^ Institute for Molecular Medicine and Internal Medicine I, Ulm University and University Hospital Ulm, Ulm, Germany; ^4^ Center for Networked Biomedical Research on Bioengineering, Biomaterials and Nanomedicine (CIBER-BBN), Madrid, Spain

**Keywords:** aging, HSC niche, deep learning, bone marrow imaging, vessel remodeling, sinusoidal niche, arteriolar niche

## Abstract

The hematopoietic stem cell (HSC) niche is a specialized microenvironment, where a complex and dynamic network of interactions across multiple cell types regulates HSC function. During the last years, it became progressively clearer that changes in the HSC niche are responsible for specific alterations of HSC behavior. The aging of the bone marrow (BM) microenvironment has been shown to critically contribute to the decline in HSC function over time. Interestingly, while upon aging some niche structures within the BM are degenerated and negatively affect HSC functionality, other niche cells and specific signals are preserved and essential to retaining HSC function and regenerative capacity. These new findings on the role of the aging BM niche critically depend on the implementation of new technical tools, developed thanks to transdisciplinary approaches, which bring together different scientific fields. For example, the development of specific mouse models in addition to coculture systems, new 3D-imaging tools, ossicles, and *ex-vivo* BM mimicking systems is highlighting the importance of new technologies to unravel the complexity of the BM niche on aging. Of note, an exponential impact in the understanding of this biological system has been recently brought by single-cell sequencing techniques, spatial transcriptomics, and implementation of artificial intelligence and deep learning approaches to data analysis and integration. This review focuses on how the aging of the BM niche affects HSCs and on the new tools to investigate the specific alterations occurring in the BM upon aging. All these new advances in the understanding of the BM niche and its regulatory function on HSCs have the potential to lead to novel therapeutical approaches to preserve HSC function upon aging and disease.

## Introduction

Hematopoietic stem cells (HSCs) were among the first stem cell types that found important clinical applications, and they are used in the laboratory and in clinic for more than five decades. Despite the huge interest and the clinical translation, it is still nowadays not possible to culture these cells and expand them in the lab, and one of the reasons for this pitfall is the importance of the *in vivo* microenvironment, which is critical to preserving the regenerative capacity of HSCs. The physiology of the HSC niche in adult mammals is complex and strictly linked to specific cell types, soluble and circulating factors, extracellular matrix components, and a quite complex three-dimensional architecture within the bone marrow. Importantly, the niche not only is a passive substrate but also exerts active functions in preserving the regenerative capacity of adult stem cells and in instructing their differentiation into progenitors. Recently, the investigation of the HSC niche upon aging revealed many unanticipated changes in the bone marrow (BM) microenvironment, which might play important roles in determining the reduction of the regenerative capacity of aged HSCs and be strongly implied also in disease progression, ranging from leukemia to myelodysplastic syndromes and to immunosenescence. This review focuses on recent work that contributed to identify major cellular players in the HSC niche and highlights the newly reported remodeling of the niche on aging. Finally, we focus on specific techniques and new computational-based approaches that are starting to be explored also in the context of the aging of the HSC niche.

## The BM Niche Supporting HSCs

The HSC niche is organized in a complex architecture, which comprises many different cell types, extracellular matrix (ECM) components, and soluble factors all involved in regulating HSC behavior. Despite the enormous advances in the understanding of the structure, function, and contribution of the BM niche in regulating HSCs, there are still many unknown aspects that require further elucidation. The emerging view tends to identify the HSC niche not as a unique and homogenous compartment but as a collection of dynamic subsets of micro-niches where different components contribute to regulate specific HSC functions. In line with this view, novel studies based on RNA sequencing and spatial transcriptomic approaches are highlighting the importance of the complexity in cell-type composition within the bone marrow (BM) niche (1, 2). Below, we will review the major niche cell types described to display an important support function for HSCs.

### Endothelial Cells

BM endothelial cells (ECs), in collaboration with perivascular cells, form specialized microenvironments shown to be involved in the regulation of HSCs and hematopoietic progenitor cells (HPCs).

ECs have been identified as one of the biggest sources of pro-hematopoietic factors such as Angiogenin, Notch ligands Jagged1 (Jag1), Jagged2 (Jag2), Delta-like ligand 1 (Dll1), and Delta-like ligand 4 (Dll4), selectin E and, in particular arteries, are enriched in the expression of CXC chemokine ligand 12 (CXCL12) and stem cell factor (SCF) (1).

Sca1+ arterial endothelial cells (aECs) constitute more than the 23% of total BMECs and are found in arteries, arterioles, and transitional vessels (or type H vessels). These aECs present a peculiar elliptically elongated nuclear shape and express high levels of vascular endothelial-cadherin (VE-Cad) and Zonulin-1 (ZO1). Type H vessels, composed by ECs highly expressing CD31 and Endomucin (CD31^hi^ and Emcn^hi^ ECs), are a specific subset of capillaries exclusively localized in the endosteal region of the BM and promote angiogenic growth and osteogenesis by providing signals to osteoprogenitor cells (3). Like type H vessels which are exclusively endosteal-localized, the most abundant fraction of arteries in the BM is in close to the endosteum. Both type H vessels and arterioles display low permeability, preserving HSCs from the exposure to high levels of reactive oxygen species (ROS) (4). Taking advantage of whole-mount histological approaches combined with mathematical modeling, arteries and rare neural glial antigen 2-positive (NG2+) perivascular cells in proximity of the endosteum have been identified as the main niche cell types promoting HSC quiescence and involved in HSC retention within the BM (5, 6).

Most BMECs are Sca1^-^ sinusoidal endothelial cells (sECs) and constitute type L vessels, which are characterized by low expression of CD31 and Emcn (3) and display a high permeability. sECs are associated with HSC mobilization, promoting their activation, and providing an exclusive area for mature leukocyte trafficking. In proximity of sinusoids, ROS levels are increased compared to arteriolar areas, and this feature enhances the migration capacity of HPCs (4). Intriguingly, sinusoids have been reported also as a specific localization site for non-dividing HSCs. Indeed, deep imaging of the BM showed that around 85% of Ki67-α-catulin-GFP+ c-kit+ HSCs are located within 10 μm from sinusoids, while Ki67+ α-catulin-GFP+ c-kit+ HSCs are mainly localized in proximity of the endosteum (7), suggesting the involvement of the sinusoidal niche in promoting HSC quiescence. This apparent discrepancy observed by Acar et al. compared to previous work identifying the peri-arteriolar/endosteal niche as the major site preserving HSC quiescence (5) can be explained considering that the staining used to identify HSCs by Kunisaki and colleagues (CD150+ CD48- CD41- lineage cells) differs from the one used by Acar and colleagues (that takes advantage of the α-catulin^GFP^ mouse model to identify HSCs as α-catulin-GFP+ c-kit+ cells). Moreover, despite the same post-imaging process used for evaluating the distance of HSCs from arteries and sinusoids, the bones used to perform the analysis, the protocol used to perform the staining, and the imaging techniques are not the same, while both authors rely on Ki67 to identify the non-dividing fraction of HSCs. Overall, the data might suggest that both arteries and sinusoids can be a preferential site for quiescent HSCs, hinting at the possible existence of different strategies played by arterial and sinusoidal ECs to promote HSC quiescence.

### EC-HSC Interaction: Focus on Notch Signaling

The direct interaction between ECs and HSCs is important to maintain and expand the HSC pool by triggering Notch activation in stem cells (8). Notch signaling is a fundamental player in the specification of HSCs during development (9, 10) and also in the regulation of adult HSCs where it is known that Notch signaling activation maintains HSC self-renewal potential (11), while its inhibition impairs HSC maintenance (12).

ECs express many Notch ligands (1, 8). For example, Jag1 and Jag2 are expressed in ECs upon angiogenic stimuli (8). The endothelium-specific knockout of Jag1, while not affecting the vascular system, exhausts the HSC pool and impairs HSC repopulation ability after transplantation and BM reconstitution after myeloablation. These data strongly indicate a key role of endothelial Jag1 in regulating HSC quiescence and self-renewal (8, 13). Endothelial Jag2 does not influence HSC homeostasis but plays a key role in regulating HSC function after myeloablation. Specifically, the deletion of Jag2 in endothelial cells causes a fast HSC exhaustion after both 5-fluorouracil (5FU) treatment and γ-irradiation (14).

The endothelial-specific inducible knockout of Dll1 does not affect any hematopoietic populations, while the endothelium-specific inducible deletion of Dll4 causes the expansion of myeloid progenitors and the reduction in the frequency of common lymphoid progenitors (CLPs), indicating that endothelial Dll4 expression regulates lymphoid lineage differentiation (1).

Of note, Notch signaling regulates HSC function also by promoting EC regeneration. The endothelium-specific deletion of the Notch1 transcriptional activation domain (TAD) in mice causes a severe reduction of HSCs and progenitor cells in BM after myelosuppression, due to increased apoptosis of ECs. The increased apoptotic rate is linked to the EC insensibility to HSC- and HSPC-dependent Angiopoietin1 (Ang1) stimulation. In control conditions, Ang1 triggers Tie2 activation, which reinforces Notch signaling in ECs and enhances Notch ligand expression, thus improving the HSC-dependent bone marrow repopulation after injury (15).

### Mesenchymal Stromal Cells and Perivascular Cells

Mesenchymal stromal cells (MSC) are rare non-hematopoietic BM cells characterized by the ability to form multipotent self-renewing mesenspheres and to self-renew in serial transplantations. These cells are identified by the expression of the intermediate filament nestin (Nes), and this aspect has been used to generate mouse models to study these cells and their contribution to the regulation of HSCs. Different groups developed similar mouse models using the Nestin gene to express the Cre recombinase and trace MSCs (NesCre mice). However, these mouse models do not overlap precisely and they were shown to target different cell types [see ref. (16, 17) for an extensive review]. Further, of Frenette’s group derived from NesCre mice a specific mouse model expressing the green fluorescent protein (GFP) under the control of the regulatory elements of the nestin promoter (Nes-GFP+ mice) (18). Based on GFP expression levels, Nes-GFP+ cells can be classified into rare Nes-GFP^bright^ cells, exclusively localized at arteries, and into more abundant Nes-GFP^dim^ cells, prevalently associated with sinusoids (5). Nes-GFP cells are innervated by noradrenergic nerve terminals and respond to this stimulation by retaining HSCs into the BM and promoting HSC and progenitor cell homing by secreting CXCL12, c-kit ligand (c-kitL), interleukin-7 (IL7), angiopoietin-1 (ANG-1), and osteopontin (OPN) (18).

Pericytes are perivascular cells displaying mesenchymal stem cell features, which have also been described as niche-supporting cells. Classically, pericytes have been divided into NG2+ cells, shown to overlap with Nes-GFP^bright^ MSC (19) and into leptin receptor-positive (LepR+) cells (20), largely coinciding with the CXCL12-abundant reticular (CAR) cell population and expressing CXCL12 and SCF (21). NG2+ pericytes are a rare cell population, mainly localized at arteries and arterioles and promoting HSC quiescence (5). LepR+ cells are mainly associated with sinusoids (6, 21, 22) and have been shown to control the stem cell pool size through CXCL12 (6) and HSC mobilization through SCF secretion (23). A recent work based on scRNA-seq data analysis deciphered and highlighted the existence of an additional level of complexity in perivascular cell organization and in their supportive function. Clustering analysis defined NG2+ cells as NG2+ and Nes+ MSC hierarchically located at the apex of differentiation into CAR cells, osteoblast, and fibroblast. Of note, CAR cells appear to include both Adipo-CAR cell population, highly expressing leptin receptor (LepR), and Osteo-CAR cells, highly expressing osterix (Sp7) and displaying low LepR levels. Interestingly, these two CAR cell subtypes contribute to HSC regulation by different cytokine secretory patterns, and due to their distinct localization, HSC function is distinctly influenced based on the specific localization within the BM niche (2).

### Osteolineage Cells

Osteoblasts and spindle-shaped N-Cadherin+ (N-cad+) osteoblastic cells are located into the trabecular bone region of the endosteum and were the first cells identified to functionally support HPCs (12, 24). Osteoblast-secreted ANG-1 (25) and OPN (26) maintain the HSC pool by promoting HSC quiescence, while parathyroid hormone (PTH) promotes HSC expansion through a Jag1-dependent activation of Notch signaling in HSCs (12). Osteoblast conditional ablation by ganciclovir-dependent activation of the herpes virus thymidine kinase (TK) gene under the control of a 2.3-kb fragment of the rat collagen 1 type I promoter (Col2.3TKmice) leads to a block in hematopoietic lineage progression with a reduction in lymphoid, erythroid, and myeloid progenitors, subsequently followed also by HSC depletion in the BM (27). Osteoblast-specific deletion of CXCL12 in mice showed that this cell component of the endosteal niche is the main effector in influencing lymphoid differentiation (6). Further, the *in vivo* lineage tracing of N-cad+ bone marrow stromal progenitor cells demonstrated that this supportive progenitor population contributes to osteoblast, adipocytes, and chondrocytes, which maintain the most quiescent HSC fraction by providing SCF and by protecting them from chemotherapeutic stress (28).

Interestingly, the endosteal peri-arteriolar niche is recently emerging as a specific lymphoid differentiation-promoting site. Indeed, in a very recent work Shen and colleagues demonstrated that peri-arteriolar LepR+ Osteolectin+ osteoblast progenitor cells promote CLP expansion and differentiation by secreting SCF, as the specific deletion of SCF from these cells strongly reduces CLP frequency in BM (29).

### Adipocytes 

The bone marrow adipose tissue (BMAT) represents 10% of the total body adipose tissue, and, interestingly, BMAT strongly differs from white adipose tissue (WAT) and brown adipose tissue (BAT). The BMAT transcriptomic profile clusters apart from WAT and BAT. Additionally, BMAT displays a higher glucose uptake and a decreased insulin responsiveness (30). BMAT is one of the most affected compartments upon aging, expanding up to occupy 50% of the BM cavity (31). Traditionally, adipocytes are considered negative regulators of the BM microenvironment and HSC function, in contraposition with osteoblasts which exert a positive function on HSC (32). Recent evidence suggests a novel and positive role for adipocytes in promoting HSC maintenance. Mattiucci and colleagues demonstrated that BM adipocytes are closely related to BM-MSCs rather than to other adipocyte populations (such as the subcutaneous adipose tissue population) and that these cells support HSC survival by expressing cell-specific cytokines, like interleukin 3 (IL3), and other MSC-overlapped cytokines (33). In line with this finding, Zhuo and colleagues showed that SCF adipocyte-specific ablation reduces mouse survival by causing HSC deficiency after myeloablation, indicating a positive role of adipocytes particularly in promoting hematopoietic reconstitution after myeloablation (34).

### β-Adrenergic Sympathetic Stimulation

The sympathetic nervous system (SNS) in the bone marrow has been shown to innervate both arteries and peri-arterial Nes-GFP+ stromal cells (5). The SNS innervation by β2 and β3 adrenergic receptors (ADR) plays a key role in the circadian mobilization of HSCs. CXCL12 is the major chemokine regulating HSC mobilization and displays an inverse pattern with HSC circadian mobilization. Interestingly, isoprenaline (a non-selective β-adrenergic agonist) treatment of bone marrow stromal cell line reduces CXCL12 expression levels, acting through the regulation of the levels of the transcription factor Sp1, and BM denervation critically alters CXCL12 circadian fluctuation in mice (35). CXCL12 levels are regulated by β3- but not β2-ADR, as both the selective β3-agonist (BRL37344) and the selective β3 antagonist (SR59230A) respectively increase and reduce the CXCL12 expression levels in the MS-5 stromal cell line. This indicates that β3-adrenergic stimulation controls the egress of HSCs from the BM (35). On the contrary, β2-ADR stimulation is involved in the reset of the local circadian clock by upregulating the *Per1* gene in the MS-5 stromal cell line, as the β2-selective agonist (clenbuterol) treatment induces *Per1* expression in the same cell line, indicating that β2-adrenergic stimulation is involved in regulating HSC homing into the BM (36).

Sympathetic innervation has been shown also to regulate HSPC and leukocyte circadian egress and homing in mice. Murine HSPCs and leukocytes preferentially home to the BM at night while during day they are released into the systemic circulation. During the night, the parasympathetic nervous system (PNS) through cholinergic stimulation reduces the egress of HSPCs and lymphocytes from BM by buffering β3-adrenergic stimulation and increasing β2-adrenergic signal, which promotes homing of hematopoietic cells by increasing the expression of vascular adhesion molecules. Conversely, during the day the depression of the β2-noradrenergic activity promotes the β3-AR-CXCL12–dependent exit of hematopoietic cells from the BM (37).

### Megakaryocytes and Macrophages

HSC progeny is an important player in regulating HSC function, and evidence shows the involvement of megakaryocytes, macrophages, neutrophils, and regulatory T cells, among others [see ref. (38) for a detailed review].

So far, megakaryocytes (MKs) are the ones mainly implied in HSC regulation and young HSCs are often found in close proximity to MKs (39, 40), which is interestingly not observed upon aging (40, 41).

MKs’ control on HSC function is dual: it has been shown that MKs control HSC quiescence by the release of specific factors like CXCL4, as both global MKs and CXCL4 depletion cause an expansion of the HSC pool and an increase in their proliferation (39). Additionally, MKs promote HSC quiescence through TGF-β secretion, which activates the SH2 domain–containing protein tyrosine phosphatase SHP-1 (42). TGF-β also regulates HSC quiescence by promoting SMAD2/3 phosphorylation in HSCs (43). It would be fascinating to verify if these two TGF-β-mediated signalings cross talk in regulating HSC quiescence. Interestingly, MKs also play a role in promoting HSC activation and proliferation after myeloablation, as MK deletion in Pf4-cre; iDTR mice by diphtheria toxin (DTR) treatment before 5FU administration causes a severe impairment of HSC expansion and BM repopulation. Upon stress stimuli, MKs start to express fibroblast grow factor 1 (FGF1), which overcomes TGF-β signaling and promotes HSC activation and proliferation (43).

Of note, the MK and HSC interplay is not unidirectional. A quiescent subpopulation of HSCs, characterized by the expression of platelet integrin CD41 (CD41), has been identified as primed toward myeloid differentiation and strongly increases upon aging (44, 45). Deletion of CD41 in HSCs leads to hematopoietic defects with loss of HSC quiescence and insensitivity of TGF-β signaling (45), supporting the existence of a positive feedback loop between CD41+ HSCs and MKs in regulating HSC quiescence and myeloid differentiation.

Macrophages have been shown to play a key role in regulating HSC quiescence and retention in the BM. DRAC+ macrophages have been shown to regulate HSC quiescence through the activation of the TGFβ1-Smad3 pathway downstream of CD82/KAI1. CD82/KAI1 is predominantly expressed in LT-HSCs, and when knocked out, HSC proliferation increases. Depletion of DRAC (CD82/KAI1-binding partner) expressing macrophages leads to a reduction of CD82/KAI1 levels in HSCs, increased proliferation, and differentiation (46). CD169+ macrophages constitute another example of cells regulating HSC retention in the BM. In CD169-iDTR mice, it has been shown that the depletion of CD169+ macrophages after DTR treatment causes an increase of HSCs and progenitor cells in peripheral blood (47). Ablation of CD169+ macrophages in CD169-iDTR mice upon DTR administration significantly reduced not only the HSC number in the BM but also the HSC, LSK, and GMP frequencies in the spleen. In line with their function in the BM, macrophages are also responsible for the retention of HSCs into the spleen, selectively through VCAM1-mediated signaling. Knockdown of VCAM1 expression in macrophages, using siRNA targeting VCAM-1 within macrophage-avid lipidic nanoparticles, causes reduced retention of splenic HSCs, LSKs, and GMPs, without affecting HSC retention into the BM (48).

## Aging of the BM Niche: Phenotypic and Functional Remodeling

Aging is a very complex physiological process that causes substantial changes in the whole organism together with tissue-specific changes in gene expression and cell composition (49). In particular in the BM, upon aging the HSC pool is expanded, and HSCs display a skewed differentiation to myeloid progenitors (50) at the expense of the lymphoid ones (51) and an impaired regenerative potential (51). The analysis of the mitotic history of HSCs and progenitors cells upon aging highlighted that HSCs and MPPs maintain their quiescent nature in a steady state, while GMLPs increase their proliferation rate (52) in line with an increase in their self-renewal potential at the expense of differentiation. Upon aging, HSCs display also loss of cell polarity (53), an intrinsic increase in Wnt5a non-canonical signaling (54), deregulated autophagy (55, 56), deregulation of the mitochondrial unfolded protein response (57), downregulation of mitochondrial acetylation mediated by SIRT3 (58), epigenomic alterations (59–62), and increased symmetry of epigenetic division (63), indicating that aging directly affects HSC function independently from the BM niche: a phenomenon described as “intrinsic” HSC aging and extensively reviewed elsewhere (64, 65). Interestingly, transplantation of young LT-HSC into aged recipient mice induces the expansion of the stem cell pool (66) and a differentiation skewing toward the myeloid lineage (67), while transplantation of aged HSCs into young recipients has been shown to rejuvenate their transcriptomic profile, despite the poor contribution to progenitor cells and the maintenance of a myeloid differentiation bias (68). Interestingly, the transplantation of rejuvenated HSCs into an aged niche restrains their rejuvenated function (69), suggesting again that BM microenvironmental aging contributes to promote an aging-associated phenotype in HSCs. This niche-dependent aging phenotype is called “extrinsic” HSC aging [reviewed in ref. (38, 70)]. Recently, this concept has been further dissected by analyzing the contribution of the middle-aged BM microenvironment, which identified the decrease in IGF1 BM levels as an essential aging-promoting factor for both HSCs and niche cells. Restoring the IGF1 signal has been shown to rescue Cdc42 and tubulin polarity, to reduce γH2AX focus and myeloid differentiation skewing in middle-aged LT-HSCs (71). In contrast, previous reports identified in the fasting-induced decrease of IGF1-dependent stimulation of PKA activity as a key factor to promote HSC self-renewal, balanced differentiation, stress resistance, and regenerative capacities after chemotherapy in aged mice (72). This apparent disagreement can be explained considering the downstream pathways activated by IGF1. The fasting-induced IGF1-mediated pathway has been described to pass through PKA activation (72), while IGF1 effects observed upon aging promote mTOR pathway activation (71). Interestingly, the mTOR pathway is dependent on nutrients and growth factors (73), suggesting more broadly that probably there is still more to understand about the regulation of HSC function by IGF1 during aging. Upon aging, many different niche compartments undergo degeneration and remodeling, affecting, on different levels of HSC behavior and function. 

### Vascular Remodeling

The BM vascular niche is profoundly changed upon aging. Despite the preservation of the endothelial area occupancy and the overall vascular volume, the frequency of endothelial cells (ECs) is reduced during physiological aging (40). These changes not only are associated with a vascular remodeling including reduction of arteries and type H vessel density but also directly affect HSC behavior (74). 

**Figure 1:**
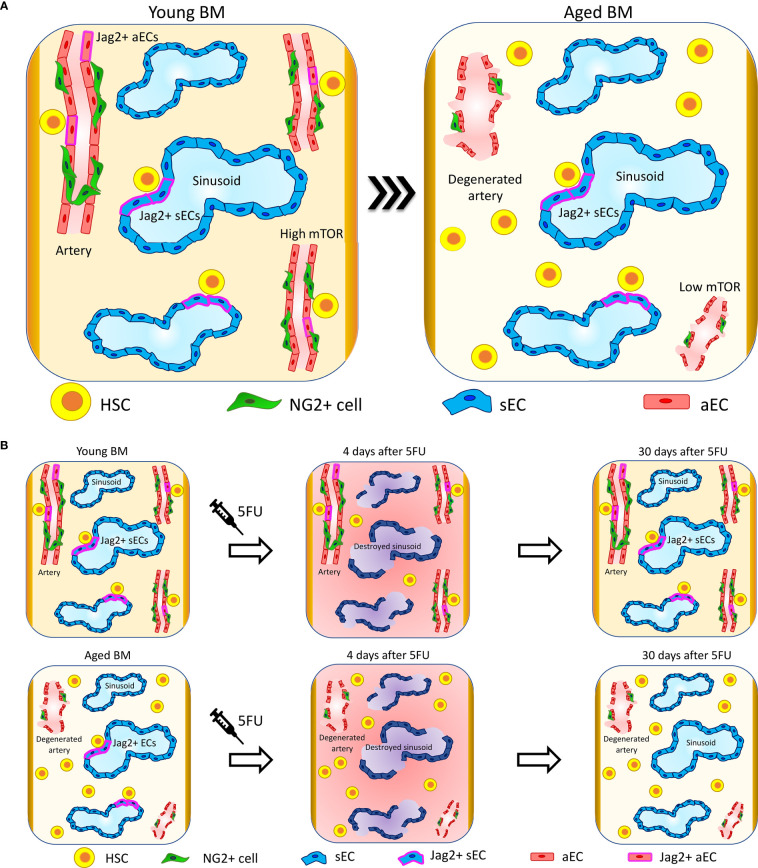
Vascular niche upon aging. **(A)** Upon aging, arteries and arterioles degenerate, changing their orientation and reducing their length (41, 75). Interestingly, specific HSC-supporting signalings are lost at the arteries. In particular, Jag2 expression is lost at arterial ECs (aECs) (40) and mTOR is downregulated (77). The sinusoidal niche preserves its structure and functionality, and sinusoidal ECs (sECs) maintain the signaling involved in the support of HSC functions. In line with these changes, HSC localization in proximity to arteries and arterioles is reduced and HSCs migrate far away. Conversely, HSCs retain their localization in proximity to sinusoids (40). **(B)** Four days after 5FU treatment, sinusoids are destroyed and there is a global increase in inflammation in both young and aged mice. Thirty days after 5FU administration, young mice recover sinusoidal vascular integrity and Jag2 expression while aged mice show only a partial restoration of sinusoidal vascular integrity and almost no recovery of Jag2. These correlate with aged HSCs localizing significantly further from sinusoids compared to control not 5FU-treated and presenting with increased clustering (40).

In a young niche, the small arterioles located into the endosteal compartment in specific association with NG2+ pericytes represent the main quiescent niche for HSCs (5). HSCs in proximity (within 20 μm of distance) to arteries and NG2+ pericytes display high retention of EdU and negative Ki67 staining. In agreement, upon induced activation or mobilization, respectively by polyinosinic-polycytidylic acid (Poly(I:C)) or G-CSF, the quiescent HSC fraction changes its localization relative to Nes+ perivascular cells and their proliferation rate increases. Interestingly, the conditional deletion of NG2+ pericytes impairs HSC long-term repopulation ability by inducing HSC cycling. This indicates that the proximity to NG2+ pericytes preserves HSCs from genotoxic insults (5). During aging, arteries and arterioles degenerate (75), decreasing their length and orientation, which becomes disorganized and not supportive anymore for the preservation of HSC quiescence (40, 76) (**Figure 1A**). Type H capillaries are also affected by aging, and their number strongly reduces over time, contrarily to sEC number which is not altered in aged mice (3). Recently, it was shown that in young mice arteries and arterioles are characterized by the expression of netrin-1 (ligand of neogenin-1), which is decreased upon aging. Neogenin-1 is exclusively expressed by quiescent HSCs and promotes the maintenance of self-renewal and quiescence. However, the aging-dependent decline of netrin-1 expression in arteries and arterioles impairs this signaling axis, leading to HSC expansion and to the reduction of their regenerative potential (76). HSCs divide rarely, and in SCL-tTAxH2B-GFP mice, the less dividing HSCs retain the pulsed histone H2B-green fluorescent protein (H2B-GFP) label *in vivo* after doxycycline (Dox) treatment and for this are defined as label-retaining HSCs (LR-HSCs) (78, 79). The analysis of LR-HSC localization in aged mice demonstrates further that quiescent HSCs in aged mice are mainly localized at sinusoids (40), in line with the expansion of the non-endosteal neurovascular niche at the expense of the endosteal niche (41). 

In young mice, the sinusoidal network occupies around 30% of the total BM volume as assessed by whole-mount histological analysis in long bones (5). Contrary to what happens to arteries and arterioles, sinusoids are largely not affected by aging and maintain the same volume occupancy, length, diameter, and orientation of the vessels, as observed in young samples (40) (**Figure 1A**).

Bone marrow ECs display a high expression of the Notch ligand Jag2 in comparison to the same cells localized in other tissues (14), and Jag2 expression is retained upon aging (40). While in young animals the endothelial-specific Jag2 knockout alters neither the proliferation rate of LT-HSCs nor their lineage composition in the BM or peripheral blood (PB) in steady state (14), the* in vivo* blockade of the endothelial Jag2 signal in aged mice causes an increase in HSC proliferation and clustering, and aged mice display a physiologic reduction of Jag2 expression at aECs (40) (**Figure 1**). It has been shown that Jag2 expression is upregulated in the recovering phase after BM myelosuppression by both 5-fluorouracil (5-FU) and lethal γ-irradiation and promotes HSPC expansion upon BM reconstitution (14). The administration of the chemotherapeutic agent 5FU, which induces in addition to myeloablation a specific sinusoidal damage, highlights critical differences in niche regeneration when comparing young versus aged mice. Indeed, in young animals there is a complete niche reconstitution and Jag2 at sinusoids is re-expressed after 30 days from treatment. In aged mice, the sinusoidal niche damage is persistent and HSC localization is affected, which results in impaired hematopoietic reconstitution and decreased overall survival after 5FU (**Figure 1B**) (40). 

As for other Notch ligands, Jag1 expression in ECs regulates HSC homeostasis and regeneration capacity (13), while EC-expressed Dll4 inhibits the activation of the myeloid transcriptional program in HSCs (1). However, the endothelial expression of these markers is not affected upon aging (40).

Notch signaling has been also demonstrated to play a key role in regulating EC proliferation and artery and type H vessel formation. The endothelium-specific overactivation of Notch signaling in aged mice increases arterial and type H vessel density, regulating HSC number. However, the endothelium-specific Notch overactivation by deletion of the *fbxw7* gene mediating Notch proteasomal degradation does not overcome the intrinsic aging of HSCs. Competitive transplantation of HSCs isolated from aged EC-specific Notch-overactivating mice do not show increased regenerative capacity nor rescuing of DNA damage accumulation (γH2AX foci) in HSCs, both classic hallmarks of intrinsic HSC aging (74).

Additional signaling pathways have been identified to regulate the functional interplay between ECs and HSCs as for example the mTOR pathway. Upon aging, ECs downregulate mTOR signaling, which induces a reduction in their support to hematopoiesis. Specific deletion of mTOR in ECs (mTOR^(ECKO)^ mice) leads to loss of α-tubulin polarity, accumulation of γH2AX foci, and change in the transcriptome of HSCs, and transplantation of young HSCs in mTOR^(ECKO)^ mice is sufficient to induce an aged phenotype in stem cells (77).

The reduced expression of heme oxygenase 1 (HO-1) in ECs and CAR cells upon aging has also been reported to impair HSCs. Upon HO-1 reduction, ECs and MSCs reduce their release of hematopoietic factors, promoting the acquisition of an aged phenotype in HSCs. Transplantation of young HO-1 wild-type HSCs into HO-1-deficient mice leads to a premature aging phenotype in transplanted cells, with the exhaustion of their regenerative potential and inability to reconstitute the BM upon secondary transplantation (80).

Collectively, this evidence indicates the importance of the vascular niche in supporting HSC quiescence, function, and stress response during aging, highlighting the importance of some specific endothelial-dependent pathways in preserving HSC regenerative potential.

### Endosteal Niche Degeneration

**Figure 2:**
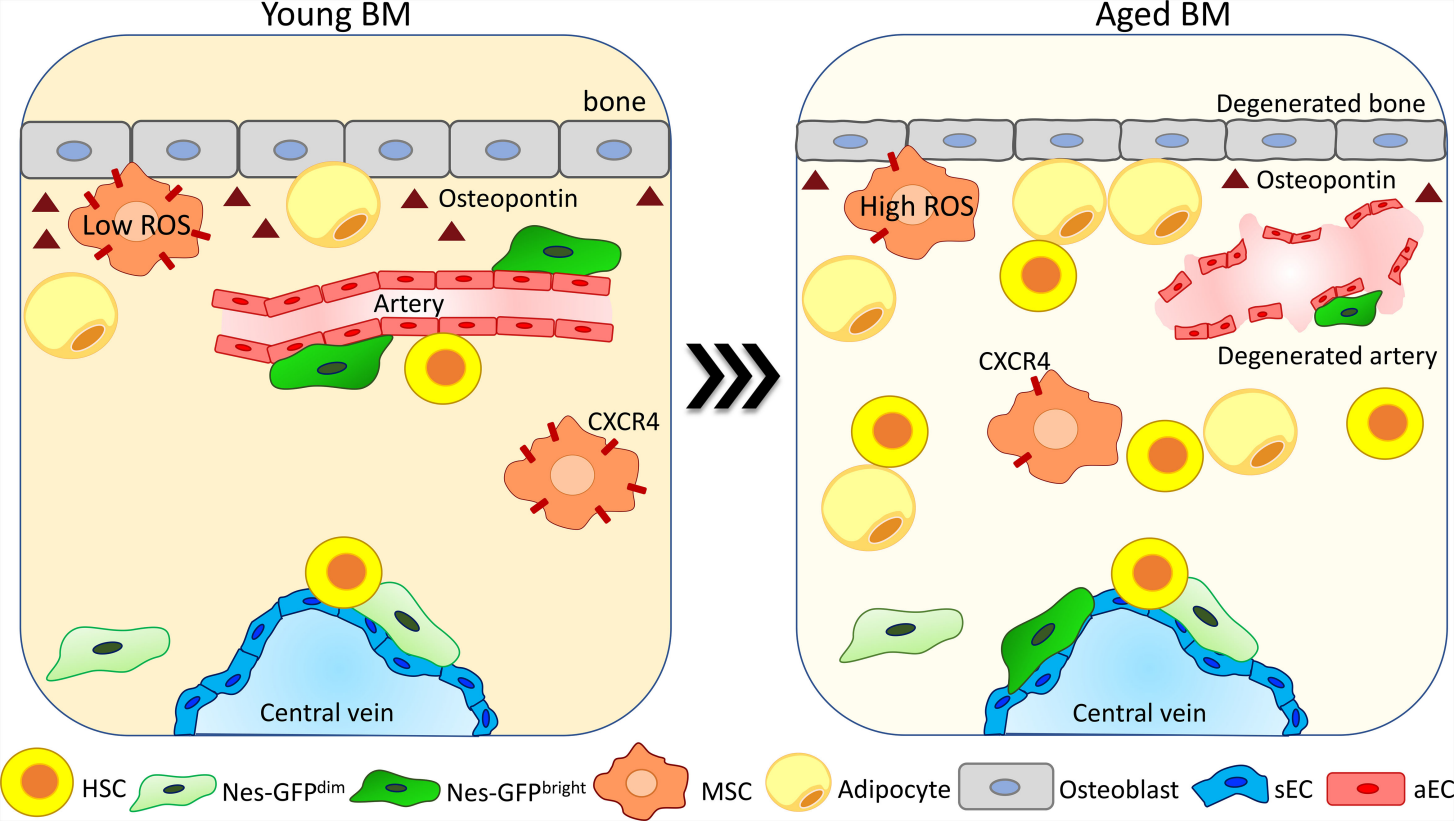
Degeneration and reorganization of the endosteal niche upon aging. The endosteal niche is one of the most affected compartments upon aging. The bone area is compromised due to a reduction in the number of osteoblasts and in osteopontin (OPN) release (26, 66, 81). Arteries and arterioles degenerate, becoming no more able to support HSC function (40, 41, 75). NES^bright^ cells change their localization from arteries moving in proximity to sinusoids, while NES^dim^ cells do not change their localization (40, 75). The MSC population is increased upon aging (81). MSCs reduce CXCR4 expression and increase ROS production, reducing their colony-forming ability and becoming less supportive for HSC maintenance (82). Adipocytes increase in number due to an enhanced pro-adipogenic differentiation of MSCs (83), promoting HSC myeloid differentiation skewing (84).

The endosteum in young mice strongly contributes to the maintenance of HSC quiescence (12, 25, 26), and upon aging the degeneration of the bone is dramatically affecting the endosteum and the arteriolar and peri-arteriolar compartment. Nes-GFP^bright^ cell frequency is reduced at the endosteum and increased in the proximity of the central vein. Moreover, these cells reduce their colony-forming ability, while the more abundant Nes-GFP^dim^ cells do not change their localization at sinusoids (75) (**Figure 2**). Interestingly, the analysis of HSC localization showed that the frequency of HSCs in proximity to Nes-GFP^bright^ cells is reduced while there are no significant alterations in HSC proximity to Nes-GFP^dim^ cells (40). In the stromal compartment, MSC number and their colony-forming activity are reduced during aging. Moreover, these cells downregulate CXCR4 expression, which leads to an increased ROS production and DNA damage accumulation (**Figure 2**). Competitive transplantation experiments showed that deletion of CXCR4 in MSCs is sufficient to drive the acquisition of an aged phenotype in young HSPCs, which show a reduced BM repopulation capacity and myeloid differentiation skewing. Interestingly, *ex-vivo* pretreatment of CXCR4-deficient MSCs with the ROS scavenger N-acetyl-L-cysteine (NAC) restores normal ROS levels in MSCs and ameliorates the phenotype of HSPCs (82). In line with these changes, intravital multiphoton microscopy analysis of the BM of young and aged mice showed the decrease of the bone matrix coupled with a reduction in the frequency of mature osteoblasts and the expansion of the mesenchymal cell population (81). Upon aging, MSCs increase IL6 and TGF-β expression and the TGF-β pathway has been demonstrated to be a key regulator of HSC aging, as its inhibition in aged HSCs reverts their enhanced platelet lineage bias and restores the lymphoid output upon transplantation (85).

During aging in human bones, the number of adipocytes is increased and correlates with a change in their milieu of secreted cytokines (86) (**Figure 2**). Studies conducted in mice showed that the expansion of adipocytes upon aging is due to a pro-adipogenic differentiation shift of osteo-adipogenic mesenchymal precursor cells, causing a reduction of hematopoietic progenitors and HSC number and repopulation capacity (83). Interestingly, a recent paper showed that the fraction of CD34+ HSPC as well as the number of differentiated myeloid cells in proximity to adipocytes is increased in the BM of aged individuals, suggesting a possible role of adipocytes in the increase of myeloid cells during aging by promoting myeloid differentiation skewing (84).

In line with changes in the adipogenic population, osteoblast (defined as CD45, Ter119, CD31, Sca1, and CD51+ cells) frequency in the BM is reduced upon aging, as well as their production of OPN (**Figure 2**). This has been shown to confer an aged phenotype to HSCs (26), and thrombin-activated OPN treatment rescues aging-related HSC phenotypes like loss of cell polarity and myeloid differentiation skewing (66). An additional study performed using OPN knockout mouse models displays also that OPN regulates the repopulation ability of aged HSCs upon transplantation (87).

Recently, new data showed that the frequency of LepR+ Osteolectin + osteogenic progenitors decreases upon aging, contributing to the reduction of the amount of CLP within the BM in the elderly. Strikingly, the reduction of LepR+ Osteolectin + osteogenic progenitors observed on aging is dependent on a change in the mechanosensing of the endosteal environment because, in concomitance with physiological or induced bone demineralization, the LepR+ Osteolectin + osteogenic progenitor population is reduced, as well as CLPs, without any significant change occurring in the frequencies of HSCs, MPPs, GMPs, MEPs, or CMPs (29). Therefore, it is likely that the aged HSC-intrinsic myeloid skewing is paralleled by a niche-dependent age-associated degeneration of the lymphoid niche.

### Sympathetic Adrenergic Signal Alterations

**Figure 3:**
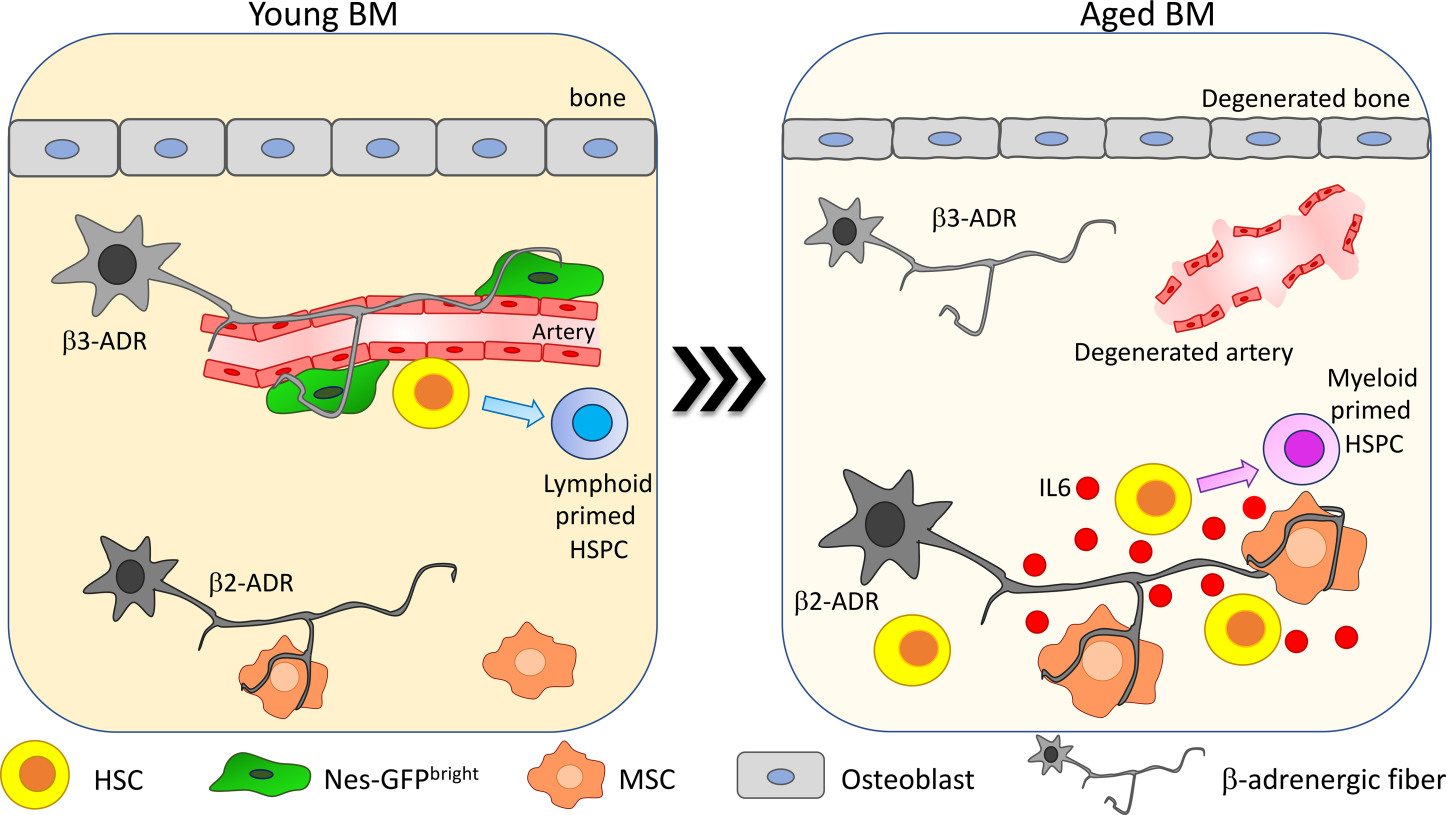
β-Adrenergic signaling alteration upon aging. β-Adrenergic innervation is strongly altered upon aging (41, 75). In young mice β3-adrenergic stimulation is predominant and promotes lymphoid differentiation while β2-adrenergic stimulation is involved in regulating myeloid differentiation. Upon aging, β2-adrenergic stimulation is increased and promotes MSC secretion of IL6, thus increasing HSC myeloid differentiation skewing.

Sympathetic adrenergic signals play a key role in regulating homing and egress of HSCs and hematopoietic cells from the BM (88), and it has been shown that the SNS innervation is strongly changed upon aging (**Figure 3**). However, there is not a clear consensus on the nature of the changes occurring to adrenergic fibers *per se* during aging, and analyses on the changes in sympathetic adrenergic innervation showed contrasting results.

In Maryanovich et al., the analysis of adrenergic fibers by staining for tyrosine hydroxylase revealed a strong and general reduction in nerve density in old BM, coupled with a reduction of perivascular Nes-GFP^bright^ cell innervation (75). Consistently, also synaptic contacts between adrenergic nerve fibers and BM-innervated cells are reduced upon aging. Taking advantage of hind-limb denervation to recapitulate the decrease of SNS stimulation occurring upon aging, it has been shown that after denervation HSCs increase their proliferation and lose their polarity for Cdc42 and tubulin and myeloid-biased CD41+ HSCs are expanded (75). Moreover, upon transplantation, HSCs collected from denervated bones display reduced engraftment compared to the non-denervated counterpart. Interestingly, β3-ADR deletion in young mice causes a premature aging phenotype and treatment with the β3-ADR agonist BRL37344 is able not only to rejuvenate HSCs by improving their engraftment potential upon transplantation and normalizing their differentiation skewing but also to rescue the acquisition of the aged phenotype in HSCs after hind-limb denervation. These findings suggest that β3-adrenergic stimulation is one of the main players in maintaining HSC regenerative potential (75).

Conversely, a more recent paper described an increase in SNS innervation upon aging. Taking advantage of the whole-mount analysis of thick-bone sections, Ho and collaborators demonstrated an increase up to 2.5 times in SNS innervation with aging in both flat and long bones, in association with a reduction of the endosteal niche. This change in adrenergic stimulation promotes myeloid differentiation skewing through the enhanced secretion of IL6 from BM stromal cells. IL6-increased secretion is triggered by the increased β2-ADR-mediated stimulation. In line with the described role for β3-adrenergic stimulation in maintaining HSC function, HSC frequency and myeloid progenitor differentiation are increased, and lymphoid differentiation is reduced in β3-ADR knockout mice (**Figure 3**). Transplantation of HSCs isolated from the progeria mouse model bearing mutation in the gene codifying for the nuclear envelope protein LaminA/C (*Lmna* ^G609G/G609G^) into healthy recipients did not recapitulate the aged phenotype observed into the progeria mouse model, while chronic treatment with the β3-ADR agonist ameliorated the aged phenotype observed in *Lmna* ^G609G/G609G^ mice, reducing the HSC frequency into the BM. Altogether, these data support a key role for the niche and in particular for β3-adrenergic stimulation in regulating the premature aging phenotype observed in the context of LaminA/C mutation (41).

Intriguingly, it has been shown that knockout of LaminA/C alters the epigenetic and chromatin architecture of HSCs similarly to what was observed in aged HSCs, which also present with very low levels of LaminA/C compared to young stem cells (59, 60). It would be therefore interesting to understand if the SNS in the BM microenvironment can impact on the epigenetic and chromatin architecture of HSCs.

Despite the absence of a consensus on the extent of the alteration of the SNS upon aging, it is clear that this niche cell type strongly affects HSCs, contributing to the aging-associated myeloid skewing.

### Inflammaging

**Figure 4:**
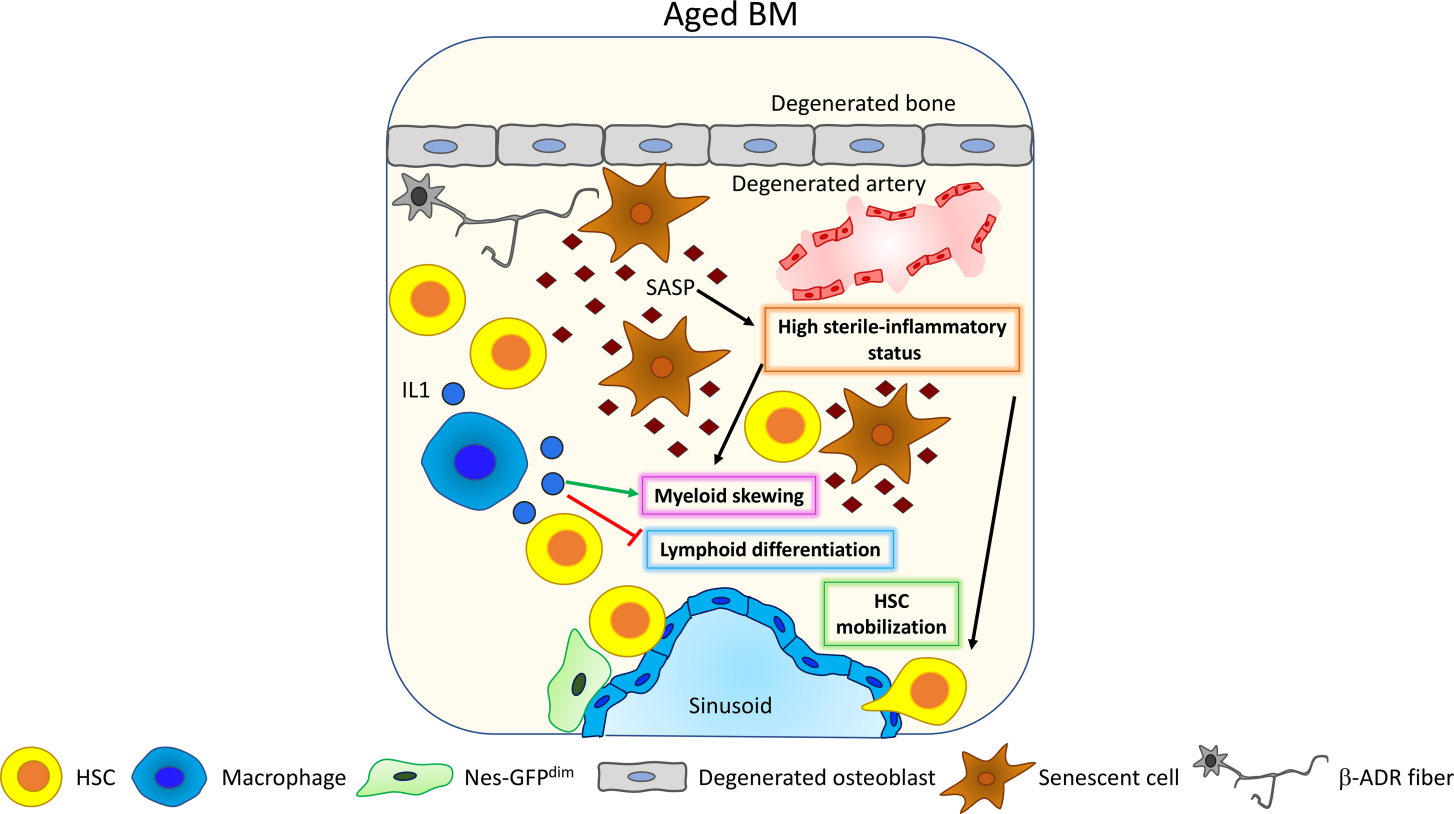
Inflammaging. Upon aging, senescent cells accumulate and acquire the so-called senescent-associated secretory phenotype (SASP) (89). SASP contains different pro-inflammatory factors that increase the general level of sterile inflammation within the BM, promoting HSC myeloid differentiation skewing (81) and HSC mobilization (91, 92). Interestingly, HSC myeloid differentiation skewing is also affected by macrophage’s activity. Upon aging, macrophages increase their release of IL1 thus promoting HSC myeloid differentiation skewing (93) and inhibiting lymphoid differentiation (94).

One of the major changes occurring upon aging in the BM is the insurgence of a low-grade inflammatory state developed in absence of any triggering infection defined as “sterile inflammation”. This systemic, chronic, and low-grade inflammation in the BM occurring during aging is termed “inflammaging” (89), and it has been postulated as one of the major stimuli promoting HSC aging and lymphoid to myeloid differentiation skewing (90). Inflammaging is mainly driven by senescent cells that accumulate upon aging (**Figure 4**). ChIP-seq analysis coupled with machine learning approaches hinted at alterations of the transcriptional and epigenetic landscape as the primary driver of the upregulation of the inflammatory response occurring upon aging (95). Senescent cells are characterized by a senescence-associated secretory phenotype (SASP), which refers to the secretion of pro-inflammatory molecules including chemokines and cytokines, bioactive lipids, and exosomes. As soon as the roles of senescent cells and SASP were identified, new classes of drugs were developed to selectively kill senescent cells (senolytics or senolytic drugs) or to inhibit the inflammatory function of the SASP components (senomorphic drugs) (96, 97). Targeting senescent cells, inflammation and SASP with senolytic drugs may represent a powerful rejuvenation tool, since a reduction of circulating levels of inflammatory cytokines has been associated with increased lifespan in several models and has also been shown to improve aged HSC function (98–101). In a recent work, Helbling and collaborators confirmed in mice the increased transcription of inflammatory cytokines, such as IL1β and IL6, and in inflammatory chemokine, such as Ccl5, Ccl6 CXCL9, CXCL10, and CXCL11, in aged BM stromal cells and endothelial cells. Interestingly, the transcriptional signature of these cells is overlapping with the signature of young BM stromal cells and endothelial cells upon lipopolysaccharide (LPS) stimulation (102). Consistently, human MSCs isolated from aged donors display a reduced colony-forming ability and a senescent-like phenotype characterized by increased β-galactosidase and SASP factors. Importantly, umbilical cord-blood (CB)-derived CD34+ HSPCs exposed to aged MSC-conditioned medium increase the expression of the inflammatory cytokines MCP1 and IL8 and reduce cell clonogenicity. This outcome is rescued if CB-derived CD34+ HSPCs are cultured in conditioned medium derived from MSCs of aged donors treated with steroids, suggesting that the main trigger in HSPC alteration is represented by the increased secretion of SASP factors from MSCs upon aging (103). Other cell types have been demonstrated to be involved in the regulation of changes in HSC and HSPC function by triggering an inflammatory response upon aging. In mouse and human aged BM, a megakaryocytic skewing with an increase in CD41+ HSCs and megakaryocytic progenitor frequency concomitant with the insurgence of an inflammatory state has been observed. Experiments in aged mice correlate this inflammatory state to an increase in activated macrophages (Mφs) with a reduced phagocytic function and increased release of IL1 (**Figure 4**). Interestingly, impaired phagocytosis in Mφs young mice, due to *alx* gene deletion, recapitulates the age-dependent myeloid skewing of HSC differentiation (81). Additionally, IL1 is sufficient to drive myeloid skewing in HSC: aged mice lacking IL1 receptor display a specific decrease of myeloid biased MPP3, while IL1 chronic administration *in vivo* expands Mac-1+Gr-1+ granulocytes, simultaneously reducing B220+ B cells (93). Consistently, treatment of aged mice with the IL1 antagonist Anakinra improves HSC repopulation ability after 5FU treatment (104). Complementary to this evidence, studies in mice (105) and in rabbits (106) demonstrate that the adipocyte-promoted myeloid expansion and IL1β production inhibit B lymphopoiesis.

Also, tumor necrosis factor alpha (TNFα) has been shown to be upregulated in HSCs upon aging, promoting myeloid differentiation skewing, HSC survival, and changes in the immunomodulatory properties through the activation of a nuclear factor-κB (NF-κB)-dependent gene program (94). Inflammaging has been shown to promote HSC mobilization (91), and Cymer and colleagues suggest the increased release of extracellular adenosine triphosphate (eATP) in the BM as a trigger for inflammation-dependent HSC mobilization (92).

Clonal hematopoiesis of indeterminate potential (CHIP) is defined as the presence in the peripheral blood of a somatic mutation with a variant allele frequency equal to or greater than 2%. CHIP is characterized by the expansion of HSC clones bearing somatic mutations, its incidence increases upon aging, and it is considered as a predisposing step to the development of hematological cancer and cardiovascular diseases (107). Proliferation and acquisition of a malignant phenotype have been linked with the presence of an inflammatory environment (107, 108). It seems likely that the pro-inflammatory changes occurring in the BM microenvironment upon aging can promote clonal hematopoiesis and its transformation to a malignancy (109). However, further experiments are needed to determine a direct cause–consequence relationship between inflammaging and CHIP.

Taken together, this experimental evidence highlights that inflammaging plays multiple and transversal roles in promoting different features of the aged-associated functional impairment of HSCs and increased cancer predisposition in the elderly.

## Tools to Investigate the Aging BM Niche

### Mouse Models

Mouse models represent one of the most important tools to study and mechanistically investigate hematopoiesis and the function of BM niche cells. For this reason, the development of new murine models has increased exponentially in the last years.

Xenotransplantation of human hematopoietic cells in mice highlighted the existence of defined but important differences in hematopoiesis and BM niche structure and supporting functions between mice and humans. However, this limitation has been partially overcome by a progressive “humanization” of several murine models, through the expression of specific human cytokines, and by the development of promising alternative strategies to mimic a human BM niche *ex-vivo* (110). In fact, the use of mouse models to study human hematopoiesis has increased exponentially in the last decades [see ref. (111) for a full list of all mouse models used for recapitulating human hematopoiesis in mice]. More in general, mouse models have been instrumental for understanding the supportive function of the niche in regulating HSCs [for an extensive review see ref (16)]. Mouse models also play a key role for deciphering the molecular mechanisms that govern many complex physiological processes, such as aging.

For example, Poulos and colleagues, using an *in vitro* coculture system, reported that young BMEC can improve aged HSPC function (112). However, only the use of a mouse model to induce the knockout of mTOR specifically in BMEC allowed the discovery of the molecular mechanism governing this supportive role of BMEC. In fact, young mice upon BMEC specific deletion of mTOR display a premature aging phenotype with increased frequency of HSCs and myeloid cells and reduced lymphoid cells. Moreover, the same mouse model allowed the analysis of the vasculature in BMEC mTOR KO animals and to point out that the observed premature aging phenotype in HSPC was due to changes in the instructive signals arising from the endothelial niche, excluding a possible role for vascular degeneration, as the vasculature of knockout mice did not manifest gross alterations (77).

An additional example of the important role of mouse models to dissect the molecular mechanisms driving the niche-dependent HSC aging is represented by OPN knockout mice.

OPN is a matrix glycoprotein secreted in the BM extracellular matrix by osteoblasts and osteocytes (113, 114). OPN levels are reduced upon aging (66), and taking advantage of the complete knockout of OPN coding gene “secreted phosphoprotein 1” (spp1), different null OPN viable mouse models have been developed (115, 116). Thanks to these mouse models, it has been possible to identify that OPN positively regulates lymphopoiesis and erythropoiesis in aged mice and directly promotes HSC regenerative capacity. Transplantation of HSCs isolated from OPN null mice into lethally irradiated mice fails to repopulate the donor BM, leading to a premature death of the transplanted mice (87). *Ex vivo* thrombin-cleaved OPN treatment of HSCs obtained from OPN null mice reverts their premature aging phenotype (66).

The work of Ho and colleagues represent an additional example of the essential role of the use of mouse models for understanding the age-dependent alteration of BM niche cells. Adrenergic stimulation is altered upon aging (75). However, different receptors participate to modulate the adrenergic response. Taking advantage of two different mouse models bearing the specific deletion of β2-ADR and β3-ADR, Ho and colleagues demonstrated a different role of adrenergic stimulation in driving HSC myeloid differentiation skewing. They observed that in aged mice, β2-ADR stimulation specifically drives myeloid differentiation skewing by a niche-dependent signal, while β3-ADR stimulation is involved in lymphoid differentiation. Aged Adrb2^-/-^ mice display a reduced frequency of myeloid progenitors, and transplantation of wild-type BM cells into Adrb2^-/-^ recipients recapitulates the megakaryocyte and platelet loss. On the contrary, β3-ADR knockout mice display a reduced frequency of lymphoid-biased HSCs in association with an increase in LT-HSC frequency and myeloid progenitors. Intriguingly, the double knockout for β2-ADR and b3-ADR does not display myeloid skewing, suggesting that the increase in adrenergic signal and the overcoming of β2-ADR signaling over the β3-ADR one are the main drivers in promoting myeloid differentiation skewing upon aging (41).

Another interesting example of the critical role of mouse models to mechanistically dissect the role of the BM niche on aging was recently provided by Frisch and colleagues (81). They demonstrated that aged mice display an impaired phagocytic activity in macrophages, in correlation with a decrease in the expression of the efferocytic receptor Alx. Taking advantage of the deletion of the tyrosine receptor Alx (117), the authors modeled a mouse with impaired phagocytosis. Young Alx knockout mice display macrophages with an impaired phagocytic activity, and this defect was sufficient to drive premature megakaryocytic skewing of HSCs (81). These data reveal the importance of mouse models as tools to decipher specific molecular pathways responsible for the interplay between stem cells and niche cells upon aging.

Mouse models can also represent a powerful tool to explore the contribution of aging in cancer development and progression. In a very recent paper, Hao and collaborators took advantage of a chronic myeloid leukemia (CML) mouse model to test how aging is affecting tumor progression in aged mice, highlighting the role of the niche in the oncogenic process (118).

### Engineering the Human BM Niche

The unique structure and architecture of BM represent a limitation for its study in humans. The inability or the difficulties in directly analyzing the whole BM in human samples stimulated the development of novel technologies to mimic and study the human BM (hBM) niche outside of its natural localization (110) also to address its role in hematological malignances. As a matter of fact, recent evidence highlighted a crucial role for the niche in disease development and leukemia expansion (119). The classical model for studying hematological malignancies is represented by xenotransplantation assays, where human hematopoietic cells are transplanted into mouse recipients. However, this type of approach excludes the possibility to investigate the signaling coming from the human niche. In these experimental setting, engraftment analysis plays a key role to define the disease and its aggressiveness. Unfortunately, mouse models sometimes fail to properly recapitulate the disease due to the murine (not human) microenvironment where the cells are transplanted. Improvements in this sense have been done by implementing immunocompromised mouse models expressing human cytokines [refer to ref (110, 120). for an extensive review]; nevertheless, this aspect requires further investigations and additional strategies are raising.

The subcutaneous implantation in mice of scaffolds supporting human niche cells represents one of the most promising strategies to mimic and study the hBM and to model hematological malignancies.

Ossicles have been described for the first time by Urist and colleagues (121) and by Freidenstein and colleagues (122) as human-derived bone formations containing in their inside structured BM (120).

Friedenstein and collaborators extensively demonstrated that freshly isolated BM cells by both trypsin digestion and bone flushing are able to generate ossicles when absorbed into porous sponges and transplanted under the renal capsule of mice (122). Subsequently, Robey and Bianco have extended the use of ossicles to model the pathogenesis of McCune-Albright fibrous dysplasia, demonstrating the relevance of this tool for clinically related investigations (123–125). Humanized ossicles can be generated by seeding hMSC into EMC-based 3D scaffolds and subcutaneously implanted in NSG-recipient mice. Abarrategi and colleagues used a porous Gelfoam^®^ scaffold composed by partially dehydrated gelatin. Human HSPCs or leukemic cells can be directly seeded into the scaffold 48 h after MSC seeding or directly injected into the mouse tail vein 4–6 weeks after the implantation of the scaffold with comparable engraftment. Once implanted, the host provides vascularization to the scaffold and is colonized with hematopoietic cells (126). Interestingly, this approach has been used to generate a humanized niche model to analyze the influence of leukemic cell remodeling of the mesenchymal niche and its effect on normal HSPC proliferation. For example, Waclawiczek and colleagues demonstrated that patient-derived AML cells impair normal hematopoiesis by influencing the release of HSPC-supporting factors by MSCs, leading to the suppression HSPC proliferation and differentiation (127).

A complementary approach to develop ossicles was described also by Reinisch and colleagues. Using this method, hMSCs are directly subcutaneously seeded into the flanks of immunocompromised NGS mice. hMSC differentiation and ossification are induced by PTH injections within 10 weeks after seeding. Normal or malignant hematopoietic cells are directly seeded into the ossicle after myeloablation (irradiation or busulfan-based chemotherapy) (128). 

Formation of LT-HSCs into ectopic niches derived from fetal bone and implanted *in vivo* under the kidney capsule requires ossification (129); however, additional approaches have been developed to mimic *in vitro* the human niche. For example, the “bone marrow–on–a–chip” represents one of such approaches (130, 131) and it consists of a poly(dimethylsiloxane) (PDMS) device coated with bone inducing materials, which is subcutaneously implanted in mice to obtain an engineered BM (eBM). The eBM can be subsequently cultured *in vitro* maintaining a functional hematopoietic system (130). However, in line with the ossicle technology, this strategy still requires the *in vivo* implantation step.

To overcome this issue in the BM-on-a-chip approach, Sieber and colleagues used a hydroxyapatite-coated Sponceram 3D ceramic scaffold to seed hMSCs and form an eBM. The similarity of the scaffold with the bone allows the formation of an eBM completely *in vitro* which functions in association to a microfluidic device to provide nutrients. Moreover, the chip system allows hHSPC seeding and differentiation, forming an eBM stable up to 28 days (131). Another very interesting approach that excludes the *in vivo* step is the *ex vivo* perfusion bioreactor model. This system consists of a hydroxyapatite ceramic scaffold inserted into a perfusion system. hMSCs are seeded into the ceramic scaffold and induced to differentiate by administering an osteogenic medium, leading to the formation of an engineered niche (eN) where CD34+ HSPCs and recombinant growth factors (SCF, TPO, FLT3-L) are subsequently added. The eN induces the expansion of phenotypic HSPCs and promotes the maintenance of stem cells, mimicking the human osteoblastic BM niche (132).

Another approach to mimic the human BM niche *in vitro* is represented by decellularized matrix scaffold [see ref. (133) for detailed information]. These scaffolds are produced by the deposition of ECM by the immortalized MSC cell line SCP-1. After the decellularization, CD34+ human HSPCs (obtained from peripheral blood after mobilization) are seeded on the scaffold. With this approach, Krater and collaborators demonstrated that these scaffolds support HSPC functionality and that they can also modulate it through integrin-mediated signaling (134).

All these strategies have proved to be extremely useful in the study of the human BM niche, and it would be intriguing to explore their potential application also for investigating aging of the human BM niche. These approaches could be useful for dissecting the contribution of specific cell types, secreted factors, and signaling pathways in impairing human HSC function over time. An engineered aged niche would offer a novel approach for defining and recreate *in vitro* the time-line cascade of events involved in aging of the BM niche, allowing not only the functional characterization of the processes involved in driving aging of the hematopoietic system but also the possibility of exploring new therapeutic approaches targeting the stem cell niche directly.

### Imaging Approaches

The BM is a densely packed tissue with a gelatinous consistence that limits its investigation and the preservation of its three-dimensional architecture by classical histological approaches. New imaging approaches have been recently developed to overcome these limitations [see ref. (135) for an extensive review].

A 3D analysis of BM architecture taking advantage of extensive BM sectioning and analysis after perfusion has been extensively used by the group of Nilsson to demonstrate that HSC localization is not random after transplantation. While progenitor and differentiated cells mainly localize in the inner marrow, HSCs prevalently localize at the endosteal area (136), requiring SCF (137) and hyaluronate (138) for their lodgment. The key aspect of this analysis is the sectioning of the BM every 3.5 μm to evaluate each stem cell only once. This approach requires extensive sectioning, and the BM 3D architecture might be easily compromised. To overcome this aspect, different strategies have been developed, like 3D-quantitative microscopy (3D-QM) and BM whole-mount histology.

3D-QM has been used by the group of Nombela-Arrieta to image the BM niche and its components (139, 140), allowing the modeling of the entire bone surface. This approach has highlighted the real abundance and complex organization of the sinusoidal network and mesenchymal reticular subsets and its maintenance upon aging (141). The same approach has been used to demonstrate that HSPCs are mainly localized at the endosteal niche in close proximity to sinusoidal and non-sinusoidal microvessels and that these cells display a hypoxic profile (142). Recently, this technique has been also applied to study the leukemic stem cell (LSC) niche. In a chronic myeloid leukemia model, the importance of CXCL12 in promoting LSC localization and clustering in close proximity of MSCs has been highlighted. Moreover, it has been shown that CXCL12 deletion in MSCs increases LSC clearance upon TKI treatment (143).

BM whole-mount histology represents one of the best strategies to preserve and analyze the BM 3D architecture. This approach allows the study of the BM niche directly in fixed bone samples by immunostaining and confocal or multiphoton microscopy imaging. This technique has been now more and more used to study different components of the BM niche and their changes upon aging (40, 41, 75). The possibility to image the BM niche from the exposed surface to the inner marrow spanning from the endosteal region to the perivascular one allowed the identification of the HSC preferential localization at arteries and endosteum in young mice (75). Moreover, this technique made possible to image and dissect the localization of rare label-retaining aged HSC at sinusoids in aged mice (40). Similarly, this technique has been used to identify the changes in the adrenergic stimulation occurring upon aging and how these are affecting HSC behavior (41, 75). However, this technique is limited by the nature of the sample and by the imaging power of the confocal or multiphoton microscopes. Samples used for whole-mount histology must be fixed in order to preserve the 3D BM structure. UV and visible light lasers used in confocal microscopy usually allow a penetrance of about 100 µm into the BM, which is further reduced in the case of combining multiple fluorophores.

To improve the possibility of resolving the composition of the BM niche by combining multiple fluorochromes together, Schroeder’s lab developed a multicolor quantitative confocal imaging approach. This technique applies to thick-bone sections from PFA-fixed long and flat bones, cleared and decalcified before the imaging process. Using a sequential staining based on primary, secondary, and tertiary antibody combinations, it is possible to image up to eight colors by confocal microscopy without linear unmixing (144). This approach has been used to map non-hematopoietic cells in the BM (145) and to demonstrate that young cycling HSCs are preferentially located in proximity to CXCL12 stromal cells and far from sinusoids and megakaryocytes (146). Interestingly, this approach has been also extended to study the functional distribution and differentiation of hMSCs seeded in ossicles (147). Moreover, this multicolor quantitative confocal imaging takes advantage of the specific image analysis software “XiT,” able to analyze large data sets and to provide internal controls for determining preferential cell localization (144). Similarly, Lucas’ lab combined the whole-mount histological approach with staining of different antibodies and the Ubc-Cre^ERT2^:Confetti mouse reporter to map the spatial segregation of myeloid progenitors during differentiation. They took advantage of the specific cytosolic localization of the Confetti reporter *vs*. the localization at the cell membrane of the antibodies to use the same color channel for staining different markers. The discrimination of the fluorescent signal localization (intracellular *vs*. cell surface) allows the clear identification of the specific cell type and its localization within the BM compartment (148).

Another powerful strategy to overcome confocal imaging limitations is represented by the use of multiphoton microscopy and by the two-photon excitation fluorescence (TPEF) microscopy. By using the near infrared light (700–1000 nm) to excite the fluorophores, it is possible to combine more colors simultaneously, reducing photobleaching in comparison to the lasers used in confocal microscopy. In addition, TPEF takes advantage of the second harmonic generation to image collagen 1 fibers in the bone (135) and the use of the infrared light for imaging increases the resolution and the penetration of the light into the samples up to a depth of 150 µm in calvaria (149). The key advantage of TPEM is its applicability both on fixed samples and for *in vivo* imaging. Currently, intravital imaging coupled with TPEM represents the best strategy to analyze the BM niche in living animals taking advantage of fluorescent reporter mouse models. The group of Von Andrian extensively used this approach to study HPC homing to BM after transplantation (150), HSC, and HSC’s progeny trafficking and homing (151–155). One of the most investigated bone for this analysis is the calvarium, because this thin skull bone does not require major manipulation prior to imaging, but it can also be applied to other bones like for example the tibiae (53, 156, 157). Of note, this imaging approach has been extensively applied to analyze young animals; however, it has been rarely used on aged mice (53, 156). This is probably due to the increased challenges of applying this technique in aged mice, which are more fragile animals, limiting the applicability of this technique. Recently, intravital imaging has been used to analyze the physiological localization of HSCs in the calvaria in relation to hypoxic areas within the BM, observing that HSCs are not found in deep hypoxic areas (158). In this example, the authors took advantage of the Mds1^GFP/+^ Flt3^Cre^ reporter mouse models to trace HSCs. The use of reporter mouse models is a key aspect of this technique, as non-viable staining cannot be performed. Other reporter mice that can be used are for example α-catulin^GFP/+^ and labeling-retaining models (146). Of note, the use of these HSC reporter lines can be combined with other reporters to image at the same time different subsets of niche cells, such as those currently used in histology as well [refer to ref (16, 17). for a detailed list]. Intravital microscopy has been also extensively used by Lo Celso’s lab to analyze the interplay between leukemic cells and the BM niche. As an example, Duarte and colleagues demonstrated that acute myeloid leukemia (AML) cells induce a massive remodeling of the endosteal BM niche by releasing pro-inflammatory and anti-angiogenic cytokines and that the degenerated endosteal niche displays a reduced capacity to support non-leukemic HSCs. Interestingly, HSC loss and the reduction of normal hematopoiesis are spatiotemporally correlated with the AML-dependent endosteal remodeling (149).

## Computational Tools to Investigate the BM Niche

Next-generation sequencing (NGS) has created a paradigm shift in medical and biological research. The advent of single-cell sequencing has further enhanced the importance of NGS and has enabled investigators to ask questions that would normally not be feasible to address *via* bulk sequencing. Single-cell sequencing methodologies enable the analysis of transcriptome, mutatome, protein–DNA interaction, and broadly the epigenome. Combined with increased statistical power and advanced analytic tools geared toward single-cell analysis, one can then look at, but not limited to, tissue heterogeneity, clonality, analysis of gene- and allele-specific expression, and single-cell level mutational analysis. For example, our groups have also applied single-cell RNA-seq and single-cell Assay for Transposase-Accessible Chromatin using sequencing (ATAC-seq) to look at transcriptional and chromatin accessibility of HSC daughter pairs (63) and allelic-specific expression associated with inactivation of chromosome X upon aging (60).

More recently, combinatorial approaches have been developed that would allow simultaneous interrogation of macromolecules (multi-omics) and spatial context of cells. This is especially highly interesting in tissues and systems with relatively higher complexity and heterogeneity. Stoeckius et al. (159) developed such a single-cell sequencing technique, which they named as Cellular Indexing of Transcriptomes and Epitopes by sequencing (CITE-seq). Using this method, one can look at both the protein markers and transcriptome profile of the same cell. By combining single-cell and spatially resolved transcriptomics where the positional information of cells is also deduced, Baccin et al. (2) mapped the molecular, cellular, and spatial compositions of distinct bone marrow niches. A more recent approach with potentially significant impact in broadening our understanding of the relationship between open chromatin and transcriptome at the single-cell level is SHARE-seq (the simultaneous high-throughput ATAC and RNA expression with sequencing) (160). Along these lines, the single-cell method has also been modified to specifically fit the needs of analysis of the niche or microenvironment in the body. NICHE-seq, developed by Medaglia et al. (161), combined fluorescent reporters, two-photon microscopy, and single-cell RNA sequencing (scRNA-seq) to infer the cellular and molecular compositions of niches. They stated that, using this technique, one can sort and analyze cells from a given region in a transgenic mouse.

In combination with the aforementioned single-cell technical advances, a seemingly obvious but still not fully exploited analytical approach that we strongly believe could significantly expand our understanding of cell biology and allow in-depth analysis of single-cell sequencing is deep learning. We have, for instance, successfully utilized deep learning to understand the positional proximity of HSCs with niche cells in the bone marrow and how, solely based on this information, one can predict whether a given HSC is obtained from a young or aged mouse (40). Our work shows the untapped potential of deep learning, even with limited number of cells, in immunofluorescence and imaging-based studies. Fortunately, most of the current single-cell technologies generate data in the range of thousands, providing a conducive platform for deep learning models that could be optimally trained and validated. In agreement with our statement and not surprisingly, Raimundo et al. (162) reported that single-cell omics has seen a surge in use of machine learning for dimensional analysis, batch normalization, classification, trajectory analysis, and inference, emanating from the flexibility and scalability of the method. Li et al. (163), for instance, used an unsupervised deep embedding algorithm to gradually removes batch effects. Yan et al. (164) discussed the potential of machine learning in single-cell sequencing, where one can use structure of cells and subpopulations with differentiation potential for stem cell therapy.

The multiome (multiple macromolecules being simultaneously interrogated in a given cell) enhances the application of machine learning by providing an additional and potentially orthogonal set of information for a given cell, thereby helping in further refinement of the deep learning model. As also stated by Li et al. (163), machine learning has been used in gene regulatory network inference or multimodal data integration based on single-cell sequencing.

## Conclusions: Where We Stand and Perspective for Therapeutic Approaches

**Figure 5:**
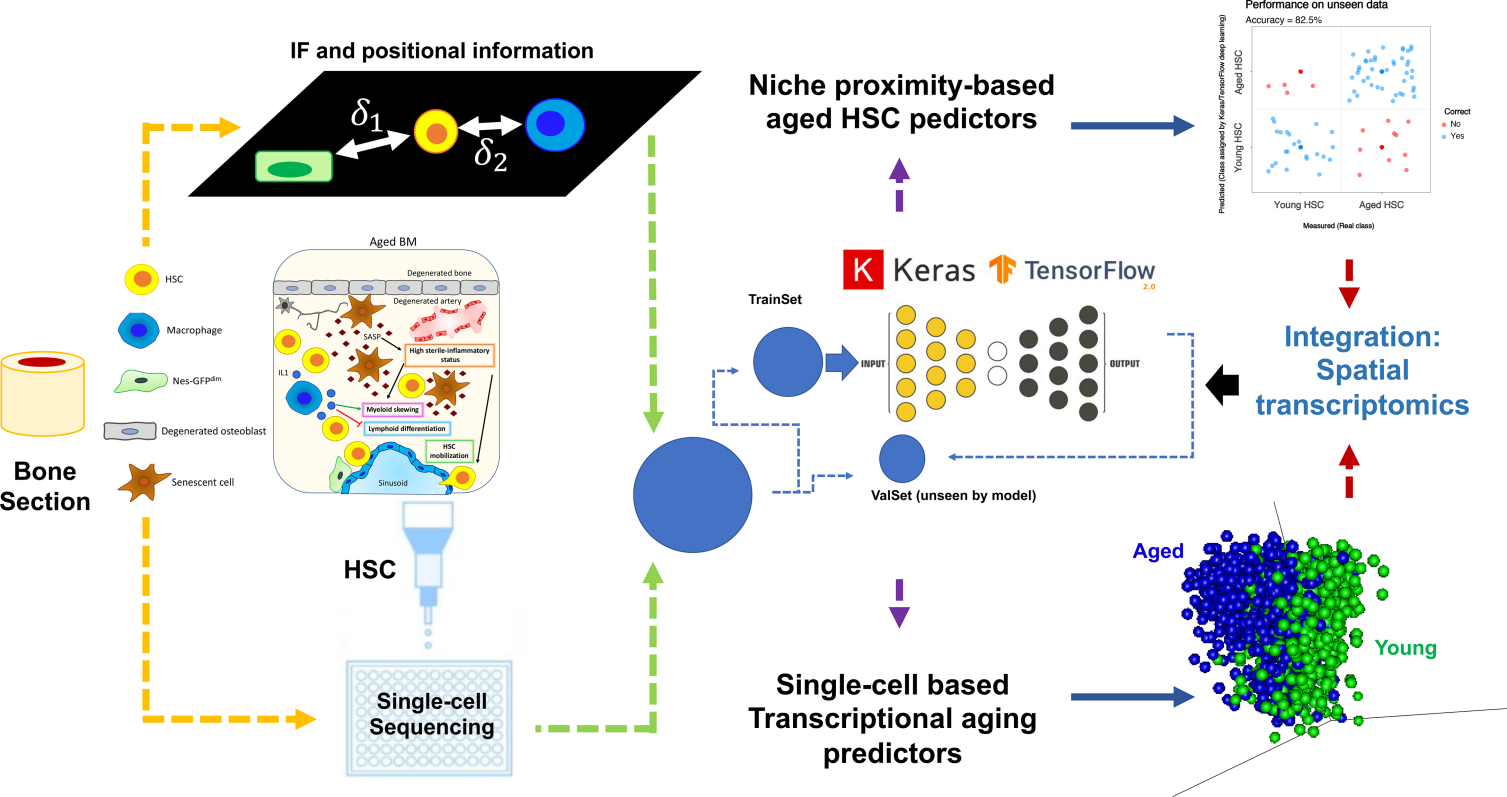
Implementation of deep learning, by integrating various datasets, to elucidate the impact of aging-mediated niche dynamics on HSC positioning and function. Here are shown two types of datasets: 1) upper part: immunofluorescence positional information showing the relative distance of HSC to niche cells and 2) lower part: single-cell sequencing of sorted HSCs. Due to the adaptability and flexibility of deep learning methodologies, both datasets are analyzed using, for instance, an R implementation of Keras/TensorFlow (165). Models are trained using a subset of the data (TrainSet) and different kernel initializations and varying numbers of hidden layers, depending on the dataset at hand. After validation of the model using a subset of data not included in the training (ValSet), prediction accuracies are calculated. For the positional information, we assess if we can predict whether a given cell is young or aged HSC purely based on their relative proximity/distance to a given niche cell and each other. As shown in our recent study (40), using such an approach, we could show ~83% prediction accuracy in defining the age status of HSCs using positional information. Using single-cell dataset and deep learning, we have also recently developed an aging signature with very high prediction accuracy (>95%; unpublished data), where the top predictors could show clear segregation of young and aged cells using principal component analysis (PCA; lower right part of the figure). We propose here that by integrating spatial transcriptomics of specific regions of a bone section (including HSCs and niche cells) and deep learning, one can perform an integrated analysis that will not only show transcriptional signatures of the cells assayed but also shed light on how the HSC-niche cell proximity and transcriptional dynamics change upon aging.

Implementation of deep learning, by integrating various datasets, is a highly promising novel approach to a long-standing question regarding niche composition, dynamics, and cell-to-cell communication. As a proof of principle, we show in **Figure 5** a model workflow on how to integrate data acquisition and analysis to elucidate the impact of aging-mediated niche dynamics on hematopoietic stem cell (HSC) positioning and functionality. Two types of datasets are shown: 1) immunofluorescence-based positional information correlating the relative distance of HSC to niche cells and 2) single-cell sequencing of sorted HSCs. As aforementioned, due to the adaptability and flexibility of deep learning methodologies, both datasets are analyzed using, for instance, an R implementation of Keras/TensorFlow (165). Models are trained using a subset of the data (TrainSet) and different kernel initializations and varying numbers of hidden layers, depending on the dataset at hand. Prediction accuracies are calculated after validation of the model using a subset of data not included in the training (ValSet). For the positional information, we assess if we can predict whether a given HSC is young or aged purely based on their relative proximity/distance to a given niche cell and each other. As shown in our recent study (40), using this approach, we could show ~83% prediction accuracy in defining the age status of HSCs using positional information. Of note, it would be interesting to refine this further, for instance, by applying spatial transcriptomics of specific regions of a bone section (including HSCs and niche cells) instead of classical single-cell sequencing.

As aging is a multifactorial biological dynamic, various types of datasets should be considered to improve our understanding of both intrinsic and extrinsic processes affecting the aging process. This also has a significant advantage in developing novel and powerful deep learning models with improved performance. By deducing the relative significance of the factors under consideration, prioritization of intervention schemes including rejuvenation and maintenance of cells of interest in an *in vivo* setting can be planned.

More broadly, investigation of the BM niche is now gaining growing attention for its new potential therapeutic and translational angle, and also other approaches, ranging from single-cell profiling, to spatial transcriptomics, to humanized niche models will all contribute to consolidate and deepen our understanding of how the BM niche supports HSC function over time. At the moment, the most general consensus view indicates the intrinsic aspects driving aging of HSC as largely fixed within the cells and with few options to be influenced by the microenvironment or by systemic rejuvenation interventions (166). However, it is interesting to underline that rejuvenation of aged HSCs proves to be beneficial to different tissues and it could be also impacting on the BM niche itself. We are just starting to explore the boundaries between intrinsic and extrinsic HSC aging and their mutual interplay. Based on the current view, it is quite likely that intervention strategies able to affect contemporarily both aspects might have a much more profound impact on hematopoiesis. Further, it would be very intriguing to explore if this combined approach targeting hematopoietic stem cell intrinsic and extrinsic aging could extend to other somatic stem cells and tissues and contribute to eventually extending lifespan and slowing aging of the whole organism.

## Author Contributions

FM, MAM, and MCF wrote the manuscript together. All authors contributed to the article and approved the submitted version.

## Funding

MCF and MAM have received funding from “la Caixa” Foundation (ID 100010434), under the agreement LCF/PR/HR20/52400014. MCF is supported by the European Research Council (ERC) under the European Union’s Horizon 2020 research and innovation program (grant agreement no. 101002453).

## Conflict of Interest

The authors declare that the research was conducted in the absence of any commercial or financial relationships that could be construed as a potential conflict of interest.

## Publisher’s Note

All claims expressed in this article are solely those of the authors and do not necessarily represent those of their affiliated organizations, or those of the publisher, the editors and the reviewers. Any product that may be evaluated in this article, or claim that may be made by its manufacturer, is not guaranteed or endorsed by the publisher.
